# Supplementary material for: LiMAx Prior to Radioembolization for Hepatocellular Carcinoma as an Additional Tool for Patient Selection in Patients with Liver Cirrhosis
Source: Cancers (Basel). 2022 Sep 21;14(19):4584. doi: 10.3390/cancers14194584 (PMC9558955; doi:10.3390/cancers14194584)
Supplement: Supplementary file 1 [file cancers-14-04584-s001.zip › cancers-1914386-supplementary.pdf]

Supplementary Information

Supplementary Table

Table S1: Causes of death during follow-up.

Patient	Causes of death	Baseline LiMAx® value (µg/kg/h)
#25	Hydropic decompensation but without relevant changes of bilirubin, albumin and INR. Relevant tumor growth with occurrence of extrahepatic tumor manifestations	363
#36	Pneumonia and new onset lung metastases	573
#40	Hepatic deterioration with an increase of total bilirubin to 4 mg/dl and hepatic encephalopathy	225
#37	Deterioration in general condition	324
#68	Deterioration in general condition	225

Supplementary Figures

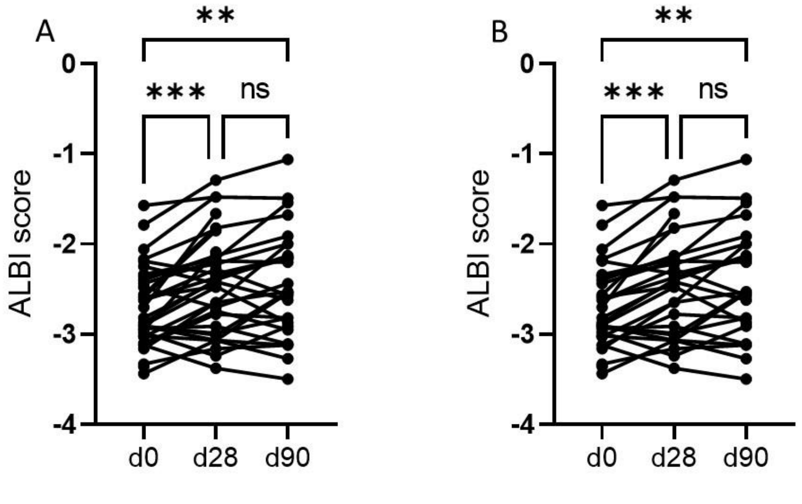

Figure S1. ALBI score at d0 (median -2.7, IQR -3.0 to -2.4), d28 (median -2.43, IQR -2.9 to -2.1) and d90 (median -2.5, IQR -2.9 to -2.0) for the entire prospective cohort. ALBI score at d0 (median -2.76, IQR -3.0 to -2.4), d28 (median -2.42, IQR -3.0 to -2.1) and d90 (median -2.53, IQR -2.9 to -2.0) only for patients with liver cirrhosis from the prospective cohort. ** = $p < 0.01$, *** = $p < 0.001$, ns = not significant.

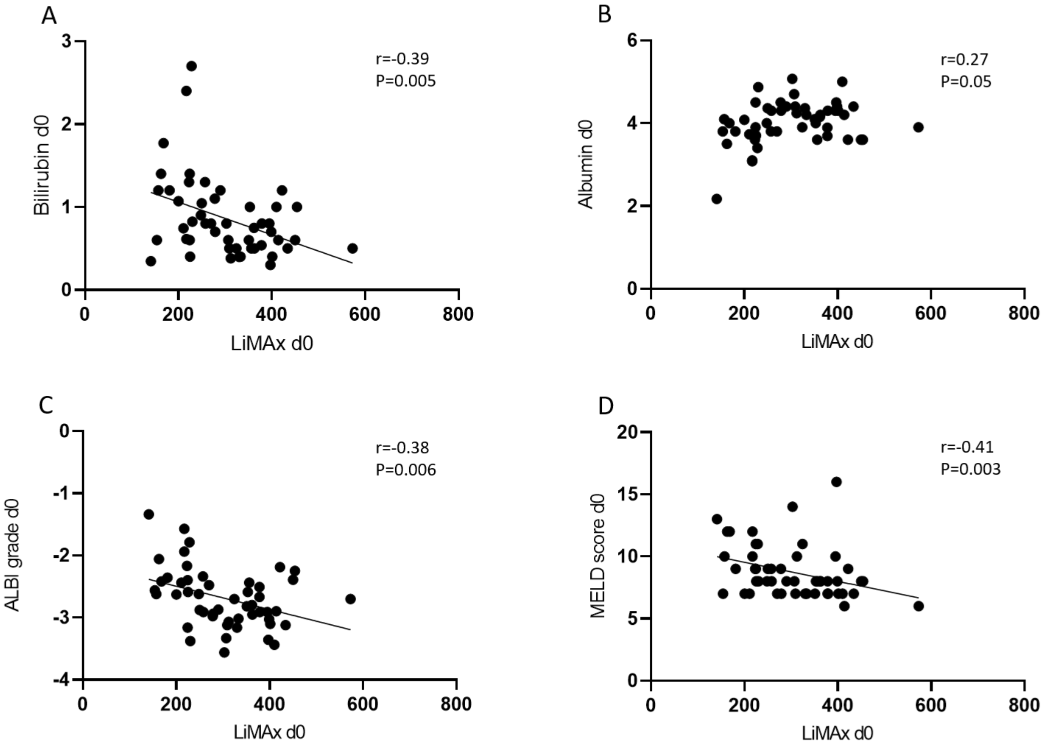

Figure S2. Correlation analysis of LiMAx® at baseline with liver function at baseline in the whole cohort–A: bilirubin r= -0.39, p = 0.005; B: albumin r=0.27, p = 0.05.; C: ALBI grade r = -0.38, p = 0.006; D: MELD score r = -0.41, p = 0.003
